# From Detection to Decision: How STIR Sequence MRI Influences Treatment Strategies for Osteoporotic Vertebral Fractures

**DOI:** 10.3390/jcm13113347

**Published:** 2024-06-06

**Authors:** Réka Viola, Siran Aslan, Mohammad Walid Al-Smadi, Dávid Süvegh, Árpád Viola

**Affiliations:** 1 Department of Psychiatry, Peterfy Sandor Hospital, 1076 Budapest, Hungary; pallareka1004@gmail.com; 2 Department of Neurotraumatology, Semmelweis University, 1081 Budapest, Hungary; drsiran.5@gmail.com; 3 Department of Neurosurgery and Neurotraumatology, Dr. Manninger Jenő National Traumatology Institute, 1081 Budapest, Hungary; smadi996@hotmail.co.uk (M.W.A.-S.); david.suvegh@gmail.com (D.S.); 4 Doctoral School of Clinical Medicine, Semmelweis University, 1083 Budapest, Hungary

**Keywords:** STIR MRI, CT, osteoporotic fracture, spine fracture, treatment planning, diagnosis protocol, surgical intervention, radiological assessment, osteoporosis, MRI effect

## Abstract

**Background/Objectives:** Osteoporotic vertebral fractures (OVFs) significantly impair quality of life. This study evaluates the impact of STIR sequence MR imaging on clinical decision-making for treating OVFs, mainly focusing on how MRI findings influence treatment modifications compared to those based solely on CT scans. **Methods:** This retrospective analysis reviewed cases from the Manninger Jenő National Traumatology Institute over ten years, where patients with suspected OVFs underwent CT and STIR sequence MR imaging. The study examined changes in treatment plans initiated by MRI findings. The diagnostic effectiveness of MRI was compared against CT in terms of sensitivity, specificity, and the ability to influence clinical treatment paths. **Results:** MRI detected 1.65 times more fractures than CT scans. MRI influenced treatment adjustments in 67% of cases, leading to significant changes from conservative–conservative, conservative–surgery, and surgery–surgery based on fracture characterizations provided by MRI. **Conclusions:** This study demonstrates that integrating STIR sequence MR imaging into the diagnostic pathway for OVFs significantly enhances the accuracy of fracture detection and profoundly impacts treatment decisions. The ability of MRI to reveal specific fracture features that are not detectable by CT scans supports its importance in the clinical evaluation of OVFs, suggesting that MRI should be incorporated more into diagnostic protocols to improve patient management and outcomes. The findings advocate for further research to establish STIR MRI as a standard osteoporosis management tool and explore its long-term benefits in preventing secondary fractures.

## 1. Introduction

The impact and complications of osteoporotic fractures cause a significant decline in the quality of life [1]. Over recent decades, an increase in sedentary lifestyles and an extended life expectancy have led to a rise in osteoporosis prevalence and, consequently, an increase in related fractures, particularly among older populations [2].

In 2000, it was estimated that osteoporosis resulted in approximately 9 million fractures globally, including 1.7 million wrist fractures, 1.6 million hip fractures, and 1.4 million clinical vertebral fractures, with 34.8% occurring in Europeans. In Europe, the disease burden from osteoporotic fractures surpasses that of most common cancers, excluding lung cancer [3]. In the United States, osteoporotic fractures are diagnosed annually in approximately 1.5 million cases, including 700,000 vertebral fractures, among other significant numbers of wrist and hip fractures [4].

The prevalence of osteoporotic fractures among women aged 50 and above in Europe varies from 18% to 26% [5]. In Hungary, the annual prevalence of osteoporotic vertebral fractures is reported at 48 per 100,000, based only on hospitalized patients [2].

The detection of osteoporotic vertebral fractures (OVFs) necessitates the use of radiological techniques such as computed tomography (CT) and magnetic resonance imaging (MRI). Choosing the appropriate imaging modality is a subject of ongoing debate due to the time and resources required for MRI scans in each suspected case. Nonetheless, MRI is recognized as the most effective method for identifying bone contusions post-trauma, indicative of trabecular microfractures, edema, and hemorrhage [6,7].

It is crucial to note that CT scans may not detect all cases of OVF [8]. Meta-analysis studies have shown that combining CT scanning with MRI significantly enhances the detection of OVFs [8,9]. The effectiveness of MRI in diagnosing spinal fractures has been well-documented, with its sensitivity rated at 100% in some studies [10]. Moreover, MRI is more adept than traditional radiographs or CT scans at revealing avascular necrosis and detecting occult fractures [11].

While STIR sequence MR imaging is known to be more sensitive in detecting fresh fractures than CT scans, no prior studies have explored the therapeutic implications or benefits of detecting additional fractures through this method. STIR MRI of the entire spine is particularly effective in identifying fractures not yet visible on CT scans [12], with its sensitivity and specificity reported to exceed 94% for diagnosing osteoporotic fractures and its predictive values surpassing 90% [13,14,15].

Post-diagnosis of an osteoporotic vertebral fracture, whether treated conservatively or surgically, there is a 34% chance of a secondary fracture occurring within a year. Secondary fractures typically present as contusions, wedges, or burst fractures [16]. The risk of an adjacent segment fracture, a new fracture occurring near a surgically treated segment, is higher among patients undergoing percutaneous vertebroplasty (PVP) than those treated conservatively [17].

The classification of osteoporotic vertebral fractures has evolved from the previously used AO classification to the Osteoporotic Vertebral Fracture Classification (OF) [18,19]. According to the OF classification, stage 1 fractures are characterized solely by edematous changes in the vertebral body, which are not visible on CT scans but can be detected as signal intensity enhancements on STIR sequence MR images.

Over ten years at the Manninger Jenő National Traumatology Institute of Péterfy Sándor Street Hospital and Outpatient Clinic, there was a notable increase in the occurrence of minor vertebral fractures related to osteoporosis (from 1 November 2008 to 31 October 2018). Our experience indicates that relying solely on CT scans is inadequate for detecting fresh fractures.

This study aims to examine the impact of STIR sequence MR imaging on the clinical decision-making process for treating osteoporotic vertebral fractures. Specifically, it seeks to demonstrate how MRI findings can modify initial treatment plans based solely on CT scans. It emphasizes MRI’s heightened sensitivity in fracture detection and its significant role in influencing the choice between conservative and surgical treatment options. Through a detailed comparative analysis of imaging results, this research aims to provide evidence that integrating STIR sequence MR imaging into the diagnostic pathway markedly enhances treatment strategies and patient outcomes, advocating for its routine inclusion in the assessment of osteoporotic fractures.

## 2. Materials and Methods

In a retrospective study conducted from 1 July 2019 to 30 November 2019, we examined a cohort of patients aged 50 and above who had sustained minor injuries resulting in vertebral fractures. The inclusion criteria were patients aged 50 or older with thoracolumbar vertebral fractures due to minor trauma, who had undergone both an entire spine CT scan and a whole spine sagittal STIR sequence MR imaging (GE HealthCare, Budapest, Hungary) within 24 h of each other. Patients with a history of tumors, ankylosing spondylitis, or diffuse idiopathic skeletal hyperostosis were excluded.

The evaluation process began with a CT scan of the whole spine, followed by an MRI of the entire spine in the sagittal STIR sequence, which also aimed to minimize the duration of patient immobility due to pain. Both radiologists and neurosurgeons independently assessed the CT and MR images. They recorded the number of new fractures identified on each modality, categorized by cervical, thoracic, lumbar, and sacral segments. Additionally, the images were reviewed to determine if the fractures were single (affecting only one vertebral body) or multiple (involving more than one vertebral body) and whether these multiple fractures were adjacent or non-adjacent.

We also assessed how the sagittal STIR sequence MRI impacted the initial treatment plan based on the CT findings, and it was divided into three groups:

1. Conservative treatment modification: If a CT scan showed a lumbar fracture alone but the MRI detected a thoracic vertebral fracture, the external orthosis was extended to cover the thoracic region.
2. Surgical intervention modification: If the CT identified an unstable fracture (AO A3, A4) without adjacent fractures, but the MRI revealed fractures adjacent to the original site, bone cement was injected using the PVP method to prevent future collapse or, depending on the case, surgical fixation was extended.
3. Conservative to surgery: If a CT indicated a stable fracture (AO A1, A2) but the MRI showed signal enhancement in the peduncles and arches of the vertebrae or a ligamentous injury, the treatment was shifted from conservative to surgical (PVP, surgical fixation).

Data were statistically analyzed using GraphPad Prism 10 software for mac (GraphPad Software, San Diego, CA, USA). We used descriptive statistics, including the mean ± standard deviation (SD), to summarize continuous variables and frequencies and percentages to describe categorical variables. The association between CT and MRI findings in diagnosing fractures was examined using the Chi-square test, with a *p*-value of less than 0.05, which was considered statistically significant.

## 3. Results

In a retrospective study spanning from 1 July 2019 to 30 November 2019, a total of 64 patients fulfilled the inclusion criteria. The cohort consisted of 64% females and 36% males, with an average age of 75.5 years (SD = 9.29). The age ranged from 52 years, the youngest participant, to 91 years, the oldest.

### 3.1. Fracture Detection and Statistical Analysis

MRI using the STIR sequence identified significantly more fractures than CT scans, detecting 266 fractures compared to 161 by CT, which represents a detection ratio of 1.65 times higher for MRI (*p*(*t*) < 0.01, *p* = 5.34, SD = 2.46, SE = 0.31). This difference in detection capability was substantiated by a paired *t*-test or one-sample *t*-test comparing the two modalities (Table 1).

### 3.2. Detailed Analysis of Fracture Types

MRI demonstrated a higher capability in identifying multiple fractures, diagnosing such cases in 91% of patients (58 cases), in contrast to 73% by CT scans (47 cases). Consequently, the proportion of single fractures identified by MRI appeared lower, with only 9% of patients (6 cases) having single fractures identified, compared to 27% by CT scans (17 cases) (Table 2). This shift in fracture detection towards multiple fractures by MRI was statistically significant, as confirmed by a Chi-square test (Chi-square value = 6.41, *p* = 0.0113). This reflects MRI’s enhanced sensitivity in detecting complex fracture patterns, rather than a deficiency in identifying single fractures.

### 3.3. Analysis of Fracture Adjacency

Among patients with multiple fractures, MRI detected adjacent fractures in 88% of cases (51/58 cases), whereas CT identified them in 74% (35/47 cases). MRI identified adjacent fractures 1.5 times more frequently than CT. However, for non-adjacent fractures, MRI identified them in 12% of cases compared to 26% by CT, with no significant difference in diagnosis rates between the two imaging types for non-adjacent fractures (Chi-square = 3.175, *p* = 0.0747) (Table 3).

### 3.4. Impact on Treatment Plans

**Figure 1:**
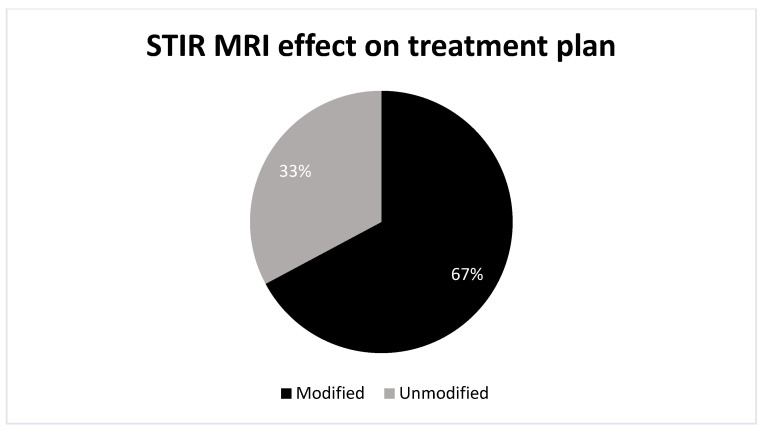
This figure demonstrates the effect of STIR MRI on the already established treatment plan based on CT. *n* = 64 patients; STIR MRI modified the treatment plan in 43 patients.

Out of the total number of patients (64 patients), MRI altered the treatment plans in 67% of the cases (43 cases), substantially impacting clinical decision-making. In 33% of the cases (21 instances), MRI findings did not conclusively affect the treatment plan that was decided on based on CT (Figure 1). Specifically, MRI led to modifications in conservative (longer brace) therapy in 58% of these impactful cases (25 instances), conversion from conservative to surgical (PVP) treatment in 30% (13 cases), and changes in surgical treatment types in 12% (5 instances) (Table 4).

### 3.5. Case Highlights

#### 3.5.1. Case 1: Conservative Treatment Modification

**Figure 2:**
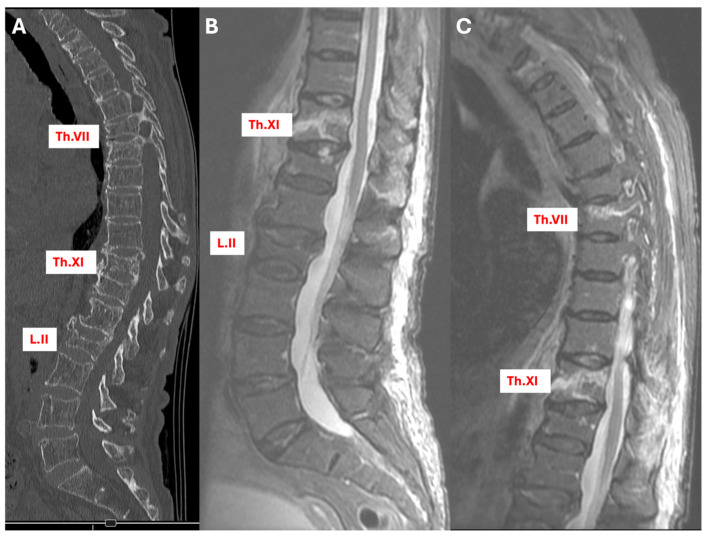
Sagittal section views of a patient with multiple fractures. (**A**) CT scan, (**B**) STIR MRI in the lumbar region, and (**C**) STIR MRI in the thoracic region, with a fresh fracture indication at Th.VII.

A patient presented with fresh fractures identified as type AO A3 at Th.XI and L.II on a CT scan, with no apparent fracture at Th.VII. However, subsequent MRI imaging revealed an additional fresh fracture at Th.VII, confirming the initial fractures and necessitating a revision of the conservative treatment plan. The result was an extension of the external orthosis to cover the upper thoracic region, providing more comprehensive support and stabilization (Figure 2).

#### 3.5.2. Case 2: Surgical Treatment Modification

**Figure 3:**
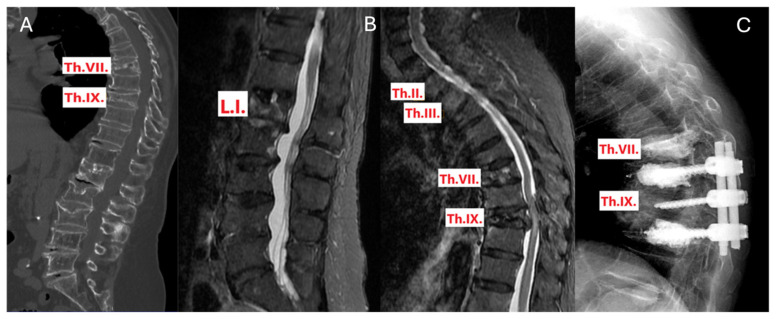
Sagittal section views of a patient with multiple fractures and subsequent intervention. (**A**) CT scan, (**B**) STIR MRI, and (**C**) X-ray, illustrating the intervention implemented following STIR MRI.

This case’s CT showed that the patient had an old fracture identified as type AO A3 at Th.VII and a fresh fracture of the same type at Th.IX, accompanied by spinal stenosis. The initial surgical plan included Th. VIII-Th. X dorsal stabilization, Th.IX laminectomy, and augmentation of Th. However, VIII and Th.X. MRI findings revealed an additional fresh fracture of type AO A1 at Th.VII. To prevent future collapse of this newly identified fracture under the added load, the surgical plan was expanded to include augmentation of Th.VII, along with the previously planned procedures (Figure 3).

#### 3.5.3. Case 3: Conversion from Conservative to Surgical Treatment

**Figure 4:**
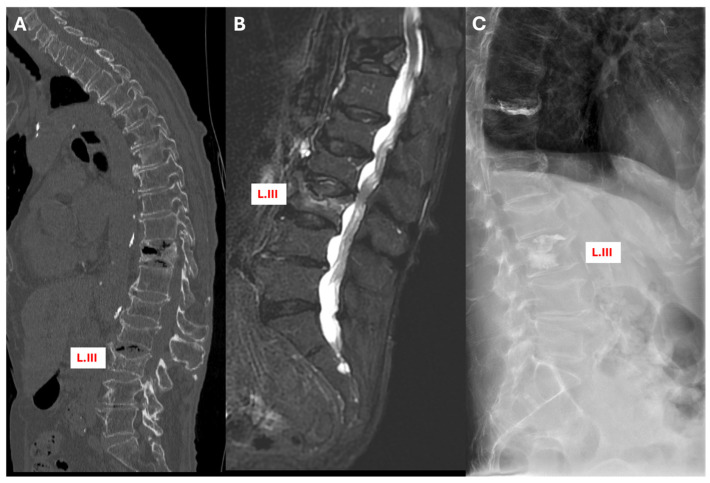
Sagittal section views of patient with multiple (fresh and old) fractures and subsequent intervention. (**A**) CT scan, (**B**) STIR MRI, with fresh fracture indication at L.III, and (**C**) X-ray, illustrating PVP in L.III.

In another instance, the CT scan failed to detect any fresh fracture at L.III, but the MRI later identified a fresh fracture at the exact location. This finding led to a significant shift in the treatment approach: the plan was changed from conservative management using a brace to cement augmentation of L.III. This adjustment addressed the fracture more effectively and prevented potential complications (Figure 4).

## 4. Discussion

This study highlights the crucial role of STIR sequence MR imaging in refining treatment strategies for osteoporotic vertebral fractures, going beyond its established diagnostic superiority over CT scans. While previous studies have extensively documented the enhanced sensitivity of MRI in detecting fractures, our research provides novel insights into how MRI findings directly influence treatment decisions and potentially improve patient outcomes.

MRI’s capability to detect 1.65 times more fractures than CT scans, as demonstrated in our results, underscores the risk of underdiagnosis when relying solely on CT imaging. This superior detection is significant for its diagnostic precision and how it translates into treatment modifications. Our findings show that in 67% of cases, MRI findings led to significant changes in the initial treatment plans based solely on CT results. This substantial rate of treatment modification highlights MRI’s potential to alter clinical perceptions and approaches to managing osteoporotic fractures.

The clinical implications of these findings are profound. For instance, MRI’s ability to reveal more complex fracture patterns can shift a treatment plan from conservative to surgical, as demonstrated in specific cases within our study. This adjustment could prevent further morbidity and improve recovery outcomes by addressing the actual extent of the injury more accurately.

Since the late 1990s, the efficacy of STIR sequence MR imaging has been recognized, with sensitivity and specificity rates exceeding 94% and both positive and negative predictive values over 90% for diagnosing fractures due to osteoporosis [6,7,8,20]. This high accuracy underpins its critical role in cases where lateral cervical CT scans fail to show abnormalities. Paula J. Richards highlights the necessity of MR imaging in such situations, especially when local neurological symptoms, intervertebral disc injuries, or spinal column damage are present, emphasizing its importance in surgical preparation. Richards recommends acquiring at least one sagittal STIR sequence MR image of the entire spine to detect non-contiguous fractures and edematous changes, supplemented by a detailed CT examination to assess for edema [21].

Building on this foundation, R.L. Williams and colleagues demonstrated that MR imaging alone could swiftly diagnose spinal instability without additional tests. Their research, which used T1W gradient echo and STIR sequence MR imaging on 22 patients with fresh fractures and unstable spines, identified both bone and soft tissue injuries, along with indirect signs of instability, like soft tissue hemorrhage. Their method relied solely on MR imaging, eschewing CT scans altogether [22].

Further emphasizing the versatility of MR imaging, J. Grünhagen and his team in 2005 used CT and MR imaging to categorize spinal injuries according to the Magerl classification. Their study of 46 fractured vertebrae in 39 patients revealed that MR imaging, using a combination of transverse and sagittal T1W spin echo, sagittal T2W spin echo, and sagittal STIR sequence, provided more detailed diagnostic information than CT alone. This included detecting more extensive bone lesions and identifying longitudinal ligament ruptures in five patients, underscoring the additional benefits of MR imaging in complex diagnostic scenarios [23,24].

Similarly, Kofi-Buaku Atsina and colleagues assessed the added value of comprehensive spine MRI following entire spine CT scans in patients with blunt injuries leading to single or multiple vertebral fractures. Their comparative analysis revealed that MRI detected additional injuries in 29 of the 156 patients, injuries not visible on CT scans. These were primarily contusions or compression fractures with minimal volume reduction, which were not considered to significantly alter the treatment plan, highlighting MRI’s ability to detect subtle yet potentially clinically insignificant findings that CT might overlook [25].

MRI, particularly the STIR sequence, is highly sensitive and can detect clinically insignificant acute bone injuries, such as bone marrow edema. This high sensitivity can identify minor or incidental findings that might not correlate with clinical symptoms or require intervention. For example, BME is a common finding in MRI scans following trauma and can persist in some cases without being clinically significant [26,27]. This oversensitivity can potentially complicate diagnosis and treatment planning if not interpreted in the proper clinical context.

These studies collectively underscore the nuanced yet significant role of MR imaging in spinal injury assessment, enhancing diagnostic accuracy and potentially influencing treatment strategies where CT scans may fall short.

In their recent article, Vratko Himič et al. explored the genetic and epigenetic factors influencing the response of patients with osteoporotic vertebral fractures to treatment. While environmental factors are challenging to characterize and evaluate accurately, understanding the genetic and epigenetic pathways involved in osteoporotic fractures offers a promising avenue for identifying useful biomarkers. These biomarkers could facilitate more precise comparisons and estimations of spinal fragility fracture burdens across different populations. The authors recommend an integrated multi-omics approach for future research, combining advances in imaging and (epi)genomics to develop practical tools for clinicians, which would improve patient management and outcomes [28].

Following the multi-approach perspective of Himič et al., involving genetics and epigenetics, and echoing Ganau. et al.’s emphasis on the importance of efficient follow-up imaging, our study highlights the crucial role of MRI in managing osteoporotic vertebral fractures [29].

Our study has several limitations that must be acknowledged. Firstly, the retrospective design and the single-institution setting limit the generalizability of our findings. The protocols and patient population specific to our institution may not reflect the broader clinical practice in other settings. Secondly, while providing valuable insights, the sample size of 64 patients may not represent the entire population of patients with osteoporotic vertebral fractures. Larger, multi-center studies are needed to validate our findings and ensure broader applicability.

Additionally, the lack of international consensus on the management of osteoporotic vertebral fractures impacts the interpretation of our results. The changes in treatment strategy described in our manuscript are based on our institution’s clinical judgment and protocols, which may differ from those in other settings. Our focus was on demonstrating the diagnostic impact of MRI STIR sequences, rather than proving the superiority of specific treatment options. The case studies in our manuscript illustrate the potential for MRI findings to alter treatment plans, but these examples may not encompass all clinical scenarios.

As mentioned, the previous research has focused on the diagnostic accuracy of MRI, which confirmed the high sensitivity and specificity of MRI for detecting spinal and associated fractures. However, our study extends this by systematically exploring how MRI’s detailed diagnostic data impact clinical decisions. Unlike earlier studies that may not have integrated clinical follow-up in their assessments, our approach involved real-time adjustments to treatment plans based on MRI findings, directly linking improved diagnostic data and clinical outcomes.

Furthermore, as Himič et al. encourage considering how genetics and epigenetics can alter the treatment plan for OVF, we support the further involvement of MRI, emphasizing its early detailed diagnostic capabilities; by incorporating MRI, which requires only 5–10 min, into the diagnostic pathway, we can achieve a more comprehensive management strategy of OVF and improve overall patient outcomes.

Given these insights, future research should aim to validate these findings across a broader demographic and investigate the long-term effects of MRI-based treatment adjustments. Establishing STIR MRI as a standard part of the diagnostic and management protocols for osteoporotic fractures could fundamentally enhance the quality of patient care, emphasizing the need for healthcare systems to adapt to these evidence-based practices.

## 5. Conclusions

In conclusion, our study not only confirms the enhanced sensitivity of MRI compared to CT but crucially demonstrates how this superior diagnostic ability can lead to significant changes in treatment plans. This contributes to a growing body of evidence that supports the essential role of advanced imaging technologies in improving the management and outcomes of patients with osteoporotic fractures.

## Figures and Tables

**Table 1:** This table presents the number of fractured vertebrae (in 64 patients) diagnosed in each spinal column level using CT and STIR sequence MRI.

Fracture Spine Level	Number of Fractures Diagnosed	
	CT	STIR MRI
C	2	7
Th	73	128
L	85	129
S	1	2
Total diagnosed fractures	161	266

**Table 2:** This table shows the number of patients with a single fracture and/or multiple fractures using CT and STIR MRI separately.

Fracture Type	Patients with Single Fracture	Patients with Multiple Fractures	Total Number of Patients
Type of Imaging			
CT	17	47	64
STIR MRI	6	58	64
Total	23	105	128 (Grand Total)

**Table 3:** This table shows the number of adjacent and non-adjacent fractures diagnosed in the patients who had multiple fractures using CT and STIR MRI separately.

Multiple Fractures Diagnosed	Patients Number with Multiple Fractures Diagnosed (%)		Total
	CT (*n* = 47)	STIR MRI (*n* = 58)	
Patients with Adjacent Fracture	35	51	86
Patients with Non-Adjacent Fractures	12	7	19
Total	47	58	105 (Grand Total)

**Table 4:** Overview of treatment modifications based on CT and STIR MRI findings, detailing changes in clinical management for 43 cases of osteoporotic vertebral fractures.

Type of Treatment Modification	Image Findings		Resultant Change	Cases *n* = 43 (Percentage)
	CT	STIR MRI		
Conservative treatment modification	Lumbar fracture alone	Additional fresh thoracic vertebral fracture	Extended external orthosis to cover the thoracic region	25 instances (58%)
Surgical intervention modification	Unstable fracture (AO A3, A4) without adjacent fractures	Additional fresh fractures adjacent to the original site	Injected bone cement using the PVP method or extended surgical fixation	13 instances (30%)
Conservative to surgery	Stable fracture (AO A1, A2)	Showed signal enhancement in the peduncles and arches of the vertebrae or a ligamentous injury	Shifted treatment from conservative to surgical (PVP, surgical fixation)	5 instances (12%)

## Data Availability

The raw data supporting the conclusions of this article will be made available by the authors on request.

## References

[1] Vokó Z., Gáspár K., Inotai A., Horváth C., Bors K., Speer G., Kaló Z. (2017). Osteoporotic Fractures May Impair Life as Much as the Complications of Diabetes. J. Eval. Clin. Pract..

[2] Péntek M., Horváth C., Boncz I., Falusi Z., Tóth E., Sebestyén A., Májer I., Brodszky V., Gulácsi L. (2008). Epidemiology of Osteoporosis Related Fractures in Hungary from the Nationwide Health Insurance Database, 1999–2003. Osteoporos. Int..

[3] Johnell O., Kanis J.A. (2006). An Estimate of the Worldwide Prevalence and Disability Associated with Osteoporotic Fractures. Osteoporos. Int..

[4] Riggs B.L., Melton L.J. (1995). The Worldwide Problem of Osteoporosis: Insights Afforded by Epidemiology. Bone.

[5] Ballane G., Cauley J.A., Luckey M.M., El-Hajj Fuleihan G. (2017). Worldwide Prevalence and Incidence of Osteoporotic Vertebral Fractures. Osteoporos. Int..

[6] Miller M.D., Osborne J.R., Gordon W.T., Hinkin D.T., Brinker M.R. (1998). The Natural History of Bone Bruises. A Prospective Study of Magnetic Resonance Imaging-Detected Trabecular Microfractures in Patients with Isolated Medial Collateral Ligament Injuries. Am. J. Sports. Med..

[7] Qaiyum M., Tyrrell P.N., McCall I.W., Cassar-Pullicino V.N. (2001). MRI Detection of Unsuspected Vertebral Injury in Acute Spinal Trauma: Incidence and Significance. Skelet. Radiol..

[8] Schoenfeld A.J., Bono C.M., McGuire K.J., Warholic N., Harris M.B. (2010). Computed Tomography Alone versus Computed Tomography and Magnetic Resonance Imaging in the Identification of Occult Injuries to the Cervical Spine: A Meta-Analysis. J. Trauma.

[9] Kepler C.K., Bogner E.A., Herzog R.J., Huang R.C. (2011). Anatomy of the Psoas Muscle and Lumbar Plexus with Respect to the Surgical Approach for Lateral Transpsoas Interbody Fusion. Eur. Spine J..

[10] Terakado A., Orita S., Inage K., Kubota G., Kanzaki T., Mori H., Shinohara Y., Nakamura J., Matsuura Y., Aoki Y. (2017). A Clinical Prospective Observational Cohort Study on the Prevalence and Primary Diagnostic Accuracy of Occult Vertebral Fractures in Aged Women with Acute Lower Back Pain Using Magnetic Resonance Imaging. Pain Res. Manag..

[11] Wang Y.-F., Teng M.M.-H., Chang C.-Y., Wu H.-T., Wang S.-T. (2005). Imaging Manifestations of Spinal Fractures in Ankylosing Spondylitis. AJNR Am. J. Neuroradiol..

[12] Blattert T.R., Schnake K.J., Gonschorek O., Gercek E., Hartmann F., Katscher S., Mörk S., Morrison R., Müller M., Partenheimer A. (2018). Nonsurgical and Surgical Management of Osteoporotic Vertebral Body Fractures: Recommendations of the Spine Section of the German Society for Orthopaedics and Trauma (DGOU). Glob. Spine J..

[13] Heynen B., Tamigneaux C., Pasoglou V., Malghem J., Vande Berg B., Kirchgesner T. (2019). MRI Detection of Radiographically Occult Fractures of the Hip and Pelvis in the Elderly: Comparison of T2-Weighted Dixon Sequence with T1-Weighted and STIR Sequences. Diagn. Interv. Imaging.

[14] Breitenseher M.J., Metz V.M., Gilula L.A., Gaebler C., Kukla C., Fleischmann D., Imhof H., Trattnig S. (1997). Radiographically Occult Scaphoid Fractures: Value of MR Imaging in Detection. Radiology.

[15] Wang B., Fintelmann F.J., Kamath R.S., Kattapuram S.V., Rosenthal D.I. (2016). Limited Magnetic Resonance Imaging of the Lumbar Spine Has High Sensitivity for Detection of Acute Fractures, Infection, and Malignancy. Skelet. Radiol..

[16] Green R.a.R., Saifuddin A. (2004). Whole Spine MRI in the Assessment of Acute Vertebral Body Trauma. Skeletal. Radiol..

[17] Medical Advisory Secretariat (2010). Percutaneous Vertebroplasty for Treatment of Painful Osteoporotic Vertebral Compression Fractures. Ont. Health Technol. Assess. Ser..

[18] Magerl F., Aebi M., Gertzbein S.D., Harms J., Nazarian S. (1994). A Comprehensive Classification of Thoracic and Lumbar Injuries. Eur. Spine J..

[19] Schnake K.J., Blattert T.R., Hahn P., Franck A., Hartmann F., Ullrich B., Verheyden A., Mörk S., Zimmermann V., Gonschorek O. (2018). Classification of Osteoporotic Thoracolumbar Spine Fractures: Recommendations of the Spine Section of the German Society for Orthopaedics and Trauma (DGOU). Glob. Spine J..

[20] Yuan W.-H., Mu-Huo Teng M., Hsu H.-C., Sun Y.-C., Chang C.-Y. (2008). Are Non-Contrast MR Images Enough for Detection of Fracture Levels Prior to Percutaneous Vertebroplasty in Patients with Osteoporosis?. Interv. Neuroradiol..

[21] Richards P.J. (2005). Cervical Spine Clearance: A Review. Injury.

[22] Williams R.L., Hardman J.A., Lyons K. (1998). MR Imaging of Suspected Acute Spinal Instability. Injury.

[23] Lenski M., Büser N., Scherer M. (2017). Concomitant and Previous Osteoporotic Vertebral Fractures. Acta Orthop..

[24] Grünhagen J., Egbers H.-J., Heller M., Reuter M. (2005). Comparison of spine injuries by means of CT and MRI according to the classification of Magerl. Rofo.

[25] Atsina K.-B., Rozenberg A., Selvarajan S.K. (2019). The Utility of Whole Spine Survey MRI in Blunt Trauma Patients Sustaining Single Level or Contiguous Spinal Fractures. Emerg. Radiol..

[26] Kumar Y., Hayashi D. (2016). Role of Magnetic Resonance Imaging in Acute Spinal Trauma: A Pictorial Review. BMC Musculoskelet. Disord..

[27] Szaro P., Geijer M., Solidakis N. (2020). Traumatic and Non-Traumatic Bone Marrow Edema in Ankle MRI: A Pictorial Essay. Insights Imaging.

[28] Himič V., Syrmos N., Ligarotti G.K.I., Kato S., Fehlings M.G., Ganau M. (2023). The Role of Genetic and Epigenetic Factors in Determining the Risk of Spinal Fragility Fractures: New Insights in the Management of Spinal Osteoporosis. Quant. Imaging Med. Surg..

[29] Ganau M., Kato S., Oshima Y. (2018). Letter to the Editor Concerning “Osteoporotic Thoracolumbar Compression Fractures: Long-Term Retrospective Comparison between Vertebroplasty and Conservative Treatment” by K. Martikos et al. [Eur. Spine J. (2018) doi: 10.1007/S00586-018-5605-1]. Eur. Spine J..

